# Episodic memory and delayed recall are significantly more impaired in younger patients with deficit schizophrenia than in elderly patients with amnestic mild cognitive impairment

**DOI:** 10.1371/journal.pone.0197004

**Published:** 2018-05-15

**Authors:** Buranee Kanchanatawan, Sookjaroen Tangwongchai, Thitiporn Supasitthumrong, Sira Sriswasdi, Michael Maes

**Affiliations:** 1 Department of Psychiatry, Faculty of Medicine, Chulalongkorn University, Bangkok, Thailand; 2 Research Affairs, Faculty of Medicine, Chulalongkorn University, Bangkok, Thailand; 3 Department of Psychiatry, Medical University of Plovdiv, Plovdiv, Bulgaria; 4 IMPACT Strategic Research Center, Deakin University, Geelong, Australia; Nathan S Kline Institute, UNITED STATES

## Abstract

**Background:**

Both amnestic mild cognitive impairment (aMCI) and schizophrenia, in particular deficit schizophrenia, are accompanied by cognitive impairments. The aim of the present study was to examine the cognitive differences between aMCI and (non)deficit schizophrenia.

**Methods:**

Towards this end we recruited 60 participants with aMCI, 40 with deficit and 40 with nondeficit schizophrenia and 103 normal volunteers. Cognitive measures were assessed with the Consortium to Establish a Registry for Alzheimer’s disease (CERAD) using the Verbal Fluency Test (VFT), Boston Naming Test (BNT), Mini-Mental State Examination (MMSE), Word list memory (WLM), Word list recall (WLRecall) and Word list recognition (WLRecognition). Data were analyzed using multivariate analyses and machine learning techniques.

**Results:**

BNT scores were significantly lower in aMCI as compared with nondeficit schizophrenia. Patients with deficit schizophrenia had significantly lower MMSE, WLM, WL True Recall and WL Recognition than aMCI patients, while WL False Recall was significantly higher in deficit schizophrenia than in aMCI. Neural network importance charts show that deficit and nondeficit schizophrenia are best separated from aMCI using total BNT score, while WLM and WL false Recall follow at a distance.

**Conclusions:**

Patients with schizophrenia and aMCI have a significantly different neurocognitive profile. Memory impairments, especially in episodic memory, are significantly worse in younger patients with deficit schizophrenia as compared with elderly patients with aMCI, while the latter show more dysnomia than patients with schizophrenia.

## Introduction

Both patients with amnestic mild cognitive impairment (aMCI) and schizophrenia show cognitive impairments. MCI is defined as a transitional cognitive state between normal ageing and early dementia and is characterized by a decline in neurocognitive functions beyond that expected by age alone [1–3]. In the Diagnostic Statistical Manual-5 of Mental Disorders, MCI is described as a “minor neurocognitive disorder” with a “significant but modest decline in cognitive function from a prior level that does not affect everyday activities and cannot be described as delirium or a major neurocognitive disorder” [4,5]. Most MCI case definitions are based on Peterson’s criteria, which include memory complaints with the absence of dementia symptoms and maintenance of daily life functions [1,2,6]. MCI may remain stable or even remit over time, although a relevant proportion of individuals with aMCI will develop Alzheimer’s Dementia[1,7,8]. A deficit in episodic memory is often observed in aMCI patients who subsequently will develop Alzheimer’s dementia [6]. The neuropsychology battery of the Consortium to Establish a Registry for Alzheimer’s disease (CERAD-NP), an instrument developed to make the diagnosis of dementia[9], was used to examine the cognitive deficits in MCI. Most studies, detected a lower total CERAD-NP score in MCI patients than in controls and lower scores on CERAD-NP subdomains including naming and semantic and episodic memory[10–16].

Schizophrenia is a disabling disorder with a heterogeneous phenotype characterized by many different symptomatic dimensions, including negative, psychotic and neurocognitive symptoms as well as symptoms of depression and anxiety. Negative symptoms, including alogia, anhedonia, apathy, social withdrawal and incongruous emotional responses, are often chronic and present between acute symptom episodes, a nosological entity named deficit schizophrenia[17]. Evidence indicates that schizophrenia and in particular deficit schizophrenia are accompanied by objective cognitive deficits as measured using the CERAD[18]. This cognitive profile in patients with deficit schizophrenia indicates impairments in immediate working memory, verbal episodic memory, recall (delayed memory tasks), verbal learning recall recognition, increased false memory creation and a more general neurocognitive defect [18]. Our data showed that deficit schizophrenia is characterized by pronounced impairments in episodic and semantic memory, rather than executive functions [19]. Moreover, the examination of patients with deficit schizophrenia and aMCI admitted to our department suggested that memory impairments could be greater in younger patients with (deficit) schizophrenia than in elderly aMCI patients. However, no research has directly compared these putative differences in cognitive profiles between individuals with (non)deficit schizophrenia and aMCI using the CERAD-NP battery.

Hence, the aim of the present study was to investigate possible differences in cognitive profile between individuals with (non)deficit schizophrenia and aMCI using CERAD tests. The a priori hypothesis was that individuals with (deficit) schizophrenia show greater impairments in episodic and semantic memory scores than patients with aMCI.

## Material and methods

### Participants

In this cross-sectional study we investigated the CERAD neuropsychological (CERAD-NP) test performance between schizophrenia and MCI patients and healthy volunteers. Thai adults of both sexes and ages 18 to 90 years were included. Participants with schizophrenia and aMCI were recruited at the Department of Psychiatry, King Chulalongkorn Memorial Hospital’s Dementia clinic, Bangkok, Thailand. Included were aMCI (n = 60) and schizophrenia (n = 80) patients. Individuals with aMCI visited our dementia clinic to investigate their subjective memory complaints. The schizophrenia patients were in a stable phase of illness and divided into 40 without (nondeficit) and 40 with deficit schizophrenia. We made the diagnosis of aMCI using Peterson criteria [19] i.e. a) subjective memory complaints, b) abnormal memory for age, c) normal general cognitive function, d) preservation of functional activities, and e) absence of dementia". Moreover, aMCI patients were included only when the Clinical Dementia Rating Scale (CDR) score was equal to 0.5 and the CDR memory component was 0.5, indicating aMCI. Schizophrenia patients were diagnosed according to DSM-IV-TR criteria and the diagnosis of deficit schizophrenia was made according to The Schedule for the Deficit Syndrome [20]. 40 healthy volunteers were recruited by word of mouth to match the schizophrenia patients for age, whereas 63 elderly controls were recruited to match the elderly aMCI patients. The latter were recruited from community senior club members, normal elderly caregivers, senior Red Cross volunteers and individuals who visited the Health Check Up Clinic, Bangkok, Thailand. All schizophrenia and aMCI patients and controls were recruited from the same catchment area, namely Bangkok province, Thailand.

We excluded aMCI and schizophrenia patients with other neurological, neurodegenerative and neuro-inflammatory disease including dementia, stroke, Parkinson’s disease, multiple sclerosis, brain tumors and epilepsy. We excluded aMCI and schizophrenia patients with other major axis-1 DSM-IV-TR diagnoses including schizoaffective disorder, major depression, bipolar disorder, substance use disorders and psycho-organic disorders. We also excluded aMCI and schizophrenia patients with major immune-inflammatory disorders including diabetes mellitus type 1, systemic lupus erythematosus, inflammatory bowel disease and chronic obstructive pulmonary disease. In addition, aMCI patients who suffered from psychosis or schizophrenia were omitted from the study as well as schizophrenia patients who developed aMCI at an older age (>60 years). We excluded controls for any disorder affecting cognition and for current and lifetime diagnoses of axis-I DSM-IV-TR mental disorders and the abovementioned neurological, neurodegenerative and medical disorders. Moreover, we excluded participants with abnormal blood tests including lower abnormal thyroid tests and BUN and vitamin B12. After considering all exclusion and inclusion criteria we recruited 103 healthy volunteers, 80 patients with schizophrenia and 60 aMCI patients.

All controls and participants with schizophrenia and aMCI as well as the guardians of all participants with schizophrenia and aMCI gave written informed consent prior to participation in this study. The study was conducted according to Thai and International ethics and privacy laws. Approval for the study was obtained from the Institutional Review Board of the Faculty of Medicine, Chulalongkorn University, Bangkok, Thailand, which is in compliance with the International Guideline for Human Research protection as required by the Declaration of Helsinki, The Belmont Report, CIOMS Guideline and International Conference on Harmonization in Good Clinical Practice (ICH-GCP). The study has been approved by King Chulalongkorn Memorial Hospital’s Research Ethic committee.

### Methods

The 80 schizophrenia patients and their age-matched controls (n = 40) were evaluated by a senior psychiatrist (KB), specialized in schizophrenia. A semistructured interview (including medical history) was conducted and diagnoses were made using the Mini-International Neuropsychiatric Interview (M.I.N.I.) [21] and DSM-IV-TR [22] criteria. The SDS was used to make the diagnosis of primary deficit schizophrenia [19]. At least 2 out of 6 features should be present for at least one year, namely restricted affect, diminished emotional range, poverty of speech, curbing of interest, diminished sense of purpose, and diminished social drive. The inclusion criteria of primary deficit schizophrenia require that the negative symptoms are not secondary to other phenomena, including extrapyramidal side effects of antipsychotics. The 60 aMCI patients and their age-matched controls (n = 63) were evaluated by a senior psychiatrist and neurologist to make the diagnosis of aMCI. Those subjects additionally underwent physical and neurological examination to exclude age-related medical and neurological pathologies. These 63 normal volunteers had a CDR score of 0 and a MMSE score of at least 24. The CDR evaluates 6 domains including memory, orientation, judgement, problem solving, social affairs, home and hobbies and personal care. An overall score 0 indicates no cognitive impairment, a 0.5 score indicates mild cognitive impairment, whilst scores of 1–3 indicate mild to severe stages of dementia [23].

All schizophrenia and aMCI patients and controls were assessed using the CERAD-NP by clinical psychologists with a master degree in mental health. They were blinded to the psychiatric diagnosis. The CERAD-NP subtests measured in this study comprise: Verbal Fluency Test (VFT), assessing semantic memory or fluency, verbal productivity, language and cognitive flexibility. Modified Boston Naming Test (BNT), measuring visual naming, confrontational word retrieval and word finding. The BNT comprises 4 measurements that vary according to frequency, namely naming of easy and highly frequency objects (BNTh), medium frequency objects (BNTm) and low frequency objects (BNTl) and their sum (BNT total). Word List Memory (WLM) measures verbal episodic memory or immediate working memory for verbal information. We measured three Trials including Trial 1 (WLM correct 1), Trial 2 (WLM correct 2), Trial 3 (WLM correct 3) and Total trials 1–3 (WLM total). Word List Recall, Delayed, true recall (WL True Recall), which measures the ability to recall (delayed recall) and verbal episodic memory recall. Word List Recall, Delayed, false recall (WL False Recall), assessing intrusion errors or false memory creation. Word List Recognition, which measures verbal learning recall recognition or verbal episodic memory-discriminability, with Word List Recognition Correct Yes response (WLRecCorrect), Word List Recognition Correct No response (WLRecNoCorrect) and their total (WLRec total). Mini-Mental State Examination (MMSE), which gives an index of a more generalized neuropsychological dysfunction and tests concentration, orientation, attention, constructional praxis, language and memory [24]. Here we use the Thai validated version [25].

### Statistics

We employed analyses of variance (ANOVA) to assess differences in scale variables between study groups and analyses of contingence tables (Χ^2^-tests) to check associations between nominal variables among groups. Correlations between two sets of scale variables were computed using Pearson’s product moment correlation coefficients. We used multivariate general linear model (GLM) analyses to delineate the effects of explanatory variables (diagnostic groups) on dependent variables (the CERAD data), while adjusting for age, sex and education. Tests for between-subject effects were subsequently used to define the univariate effects of the significant explanatory variables on the dependent variables. Hierarchical binary logistic regression analyses were employed to delineate the significant explanatory variables (CERAD data) which predict aMCI versus deficit schizophrenia and aMCI versus nondeficit schizophrenia. Odds ratios and upper-lower 95% confidence intervals were computed, while Nagelkerke values were used as estimates of effect size. The results of multiple analyses were p-corrected for false discovery rate [26]. Tests were two-tailed and a p-value of 0.05 was used for statistical significance.

In this study we employed different supervised machine learning techniques, including Linear Support Vector Machine (SVM) and Random Forest, using the Scikit-learn package of Python programming language [27]. 10-fold cross-validations with accuracy (SD) and the relative importance of the CERAD features for aMCI versus deficit schizophrenia and MCI versus nondeficit schizophrenia were computed. We evaluated the weight of the predictor variables in Linear SVM and the importance in Random Forest models. In Linear SVM, the sign of the weights is associated with the diagnostic class, while the magnitude reflects its importance. In Random Forest, the importance is calculated based on GINI importances, which measure the average gain in purity of diagnosis classes when that feature is used as criterion to split data points. We also used multilayer perceptron (MLP) Neural Network (NN) procedures to investigate more complex relationships using automated feedforward architecture models with CERAD test scores (with or without age, sex and education) as input variables, while the output layer contains the aMCI and schizophrenia groups. We considered one or two hidden layers with a variable number of nodes. Error, the rate of incorrect predictions based on training and testing sample and the partitioned confusion matrix for output categorical variables were computed as well as the area under the receiving operating curves (AUC ROC) and (relative) importance of each of input variable in sensitivity analyses. All data were analyzed using IBM SPSS windows version 24, Statistica 12, MaesStat and MATLAB.

## Results

### Socio-demographic data

Table 1 shows age, sex and education in the four diagnostic subgroups. Both schizophrenia subgroups were significantly younger than aMCI patients, while the control subjects occupied an intermediate position. These differences are inherent to the study sample selection, namely elderly subjects with aMCI (>62 years) and younger schizophrenia subjects in a stable phase of illness. Since the healthy control group consisted of a younger subsample (age-matched to schizophrenia patients) and an elderly subsample (age-matched to aMCI patients) the total control group showed an intermediate age.

**Table 1:** Socio-demographic data in normal controls, schizophrenia patients with and without deficit schizophrenia and patients with amnestic mild cognitive impairment (aMCI).

Variables	Controls ^A^	Nondeficit Schizophrenia ^B^	Deficit schizophrenia ^C^	aMCI ^D^	F/X^2^	df	p
Age (years)	56.2 (17.5) ^B,C,D^	41.3 (10.8) ^A,D^	40.9 (11.4) ^A,D^	74.8 (6.3) ^A,B,C^	71.58	3/239	<0.001
Age range (years)	20–83	20–59	19–65	62–90	-	-	-
Sex (male / female)	21 / 82 ^B,C^	22 / 18 ^A,D^	21 / 19 ^A^	16 / 44 ^B^	24.16	3	<0.001
Education (years)*	13.2 (5.0) ^C,D^	12.8 (4.2)	11.8 (4.1) ^A^	10.2 (10.2) ^A^	3.78	3/237	0.011

All results are shown as mean (SD). F: results of analyses of variance; X^2^: results of analyses of contingency tables.

* These results were adjusted for age and sex

Since the main aim of the study was to analyze the degree of cognitive impairment present in younger schizophrenia patients versus that in aMCI patients (thus elderly individuals) we have carried out GLM analyses without adjustment for age and showed these results in Figures (primary analyses) and with adjustment for age. The associations between age and CERAD test scores were computed with Pearson’s product moment correlations in normal controls. The latter study sample shows an age range of 20 to 83 years, and therefore comprises an unrestricted sample that allows us to examine the effects of age on the CERAD test scores. Table 2 shows that in normal controls, there were significant and inverse associations between age and VFT, BNT and MMSE, but not the other CERAD tests. Therefore, we have also adjusted the data for age by entering the latter as an additional explanatory variable in the analyses and show the age-adjusted data in Table 3 (secondary analyses). In patients with schizophrenia and aMCI no significant associations were detected between age and the CERAD tests.

**Table 2:** Correlation matrix between the Consortium to Establish a Registry for Alzheimer’s disease test results and age and education in healthy controls.

CERAD tests	Age	Education
VFT	-0.265 (0.007)	+0.183 (0.065)
BNT high	-0.062 (0.536)	+0.167 (0.091)
BNT medium	-0.341 (<0.001)	+0.305 (0.002)
BNT low	-0.504 (<0.001)	+0.405 (<0.001)
BNT total	-0.496 (<0.001)	+0.422 (<0.001)
MMSE	-0.321 (0.001)	+0.611 (<0.001)
WL Correct1	-0.118 (0.235)	+0.070 (0.483)
WL Correct2	-0.105 (0.290)	+0.135 (0.175)
WL Correct3	-0.086 (0.388)	+0.222 (0.024)^a^
WLM	-0.122 (0.218)	+0.159 (0.110)
WLRecall True	+0.002 (0.988)	+0.116 (0.245)
WLRecall False	-0.002 (0.988)	-0.116 (0.245)
WLrecognition Correct	-0.042 (0.670)	+0.089 (0.373)
WLRecognition Nocorrect	-0.012 (0.903)	-0.095 (0.342)
WLRecognition total	-0.067 (0.503)	+0.012 (0.908)

^a^No longer significant after p correction for false discovery rate (p = 0.072)

**Table 3:** Results of multivariate general linear model analyses with the Consortium to Establish a Registry for Alzheimer’s disease (CERAD) tests results as dependent variables and diagnosis as primary explanatory variable, while adjusting for age, sex and education. Shown are the model-generated marginal means (SE) of the z-scores of all CERAD tests.

Variables	Controls ^A^	Nondeficit Schizophrenia ^B^	Deficit schizophrenia ^C^	Amnestic mild cognitive imairment ^D^	F	df	p	Partial Eta squared
VFT	0.466 (0.093) ^B,C,D^	-0.162 (0.149) ^A,C^	-0.834 (0.149) ^A,B,D^	-0.102 (0.138) ^A,C^	20.33	3/235	<0.001	0.206
BNT high	0.077 (0.108)	-0.072 (0.173)	-0.113 (0.172)	-0.128 (0.159)	0.65	3/235	0.581	0.008
BNT medium	0.218 (0.096) ^D^	0.025 (0.154)	0.057 (0.154)	-0.134 (0.142) ^A^	1.77	3/235	0.153	0.022
BNT low	0.140 (0.084) ^D^	0.276 (0.135) ^D^	0.096 (0.135)	-0.251 (0.125) ^A,B^	2.96	3/235	0.033	0.036
BNT total	0.194 (0.085) ^D^	0.180 (0.135) ^D^	0.070 (0.135)	-0.238 (0.125) ^A,B^	3.08	3/235	0.028	0.038
MMSE	0.247 (0.087) ^B,C^	-0.154 (0.139) ^A,C^	-0.698 (0.139)^A,B,D^	0.071 (0.128) ^C^	10.88	3/235	<0.001	0.122
WLM correct1	+0.317 (0.094) ^B,C,D^	-0.364 (0.150) ^A,C^	-0.831 (0.151) ^A,B,D^	-0.028 (0.140) ^A,C^	14.60	1/236	<0.001	0.157
WLM correct2	+0.354 (0.091) ^B,C,D^	-0.312 (0.145) ^A,C^	-0.919 (0.146) ^A,B,D^	-0.022 (0.135) ^A,C^	18.76	1/236	<0.001	0.193
WLM correct3	+0.344 (0.086) ^B,C^	-0.311 (0.136) ^A,C^	-1.059 (0.138) ^A,B,D^	-0.022 (0.135) ^C^	24.59	1/236	<0.001	0.238
WLM total	+0.376 (0.086) ^B,C,D^	-0.365 (0.136) ^A,C^	-1.039 (0.137) ^A,B,D^	+0.003 (0.127) ^A,C^	25.79	1/236	<0.001	0.247
WL True Recall	+0.400 (0.090) ^B,C,D^	-0.069 (0.143) ^A,C^	-0.923 (0.144) ^A,B,D^	-0.188 (0.133) ^A,C^	22.80	3/236	<0.001	0.225
WL False Recall	-0.394 (0.090) ^B,C,D^	+0.052 (0.143) ^A,C^	+0.928 (0.144) ^A,B,D^	+0.190 (0.134) ^A,C^	22.65	3/236	<0.001	0.224
WLRecCorrect	+0.229 (0.102) ^C^	+0.026 (0.161) ^C^	-0.673 (0.162) ^A,B,D^	-0.064 (0.150) ^C^	8.00	3/236	<0.001	0.092
WLRecNoCorrect	-0.026 (0.109)	+0.065 (0.173)	-0.255 (0.175)	+0.085 (0.162)	0.87	3/236	0.456	0.011
WLRec total	+0.213 (0.102) ^C^	+0.065 (0.162) ^C^	-0.667 (0.163) ^A,B,D^	-0.064 (0.151) ^C^	7.72	3/236	<0.001	0.089

VFT: Verbal Fluency Test. BNT: Boston Naming Test (BNT) with easy and highly frequency objects (BNT high), medium frequency objects (BNT medium) and low frequency objects (BNT low) and their sum (BNT total). MMSE: Mini-Mental State Examination. WLM: Word list memory, with three Trials including Trial 1 (WLM correct 1), Trial 2 (WLM correct 2), Trial 3 (WLM correct 3) and their total (WLM total). WLRecall True: Word List Recall, Delayed, true recall. WLRecall false: Word List Recall, Delayed, false recall. WLRecCorrect: Word List Recognition Correct Yes response. WLRecNoCorrect: Word List Recognition Correct No response. WLRec total: World List Recognition total.

In normal controls, GLM analyses (age-adjusted) show significantly higher MMSE (F = 4.38, df = 1/100, p = 0.039) and BNT (F = 10.95, df = 1/100, p<0.001) scores in men than women (MMSE: 28.63 ±0.42 versus 27.65 ±0.21 and BNT 13.84 ± 0.36 versus 12.49 ±0.18, respectively). There were no significant differences in the other CERAD tests. Nevertheless, since there were differences in sex ratio between the study groups, we have used sex as a second factor in the secondary analyses. Table 1 shows that years of education (age- and sex-adjusted) was significantly lower in patients with aMCI and deficit schizophrenia as compared with controls, while nondeficit schizophrenia patients took up an intermediate position. In order to define the correlations between education and the CERAD test scores we have computed the intercorrelation matrix between the two variables in normal controls. Table 2 shows that there were significant and positive associations between education and BNT and MMSE, but no significant correlations with the other CERAD tests (after p-correction was made). However, since lower education may be a risk factor for aMCI and deficit schizophrenia (see Discussion) we have examined inter-group differences with (secondary analyses) and without (primary analyses) adjustment for education.

### VFT, BNT and MMSE score differences between the diagnostic groups

**Fig 1:**
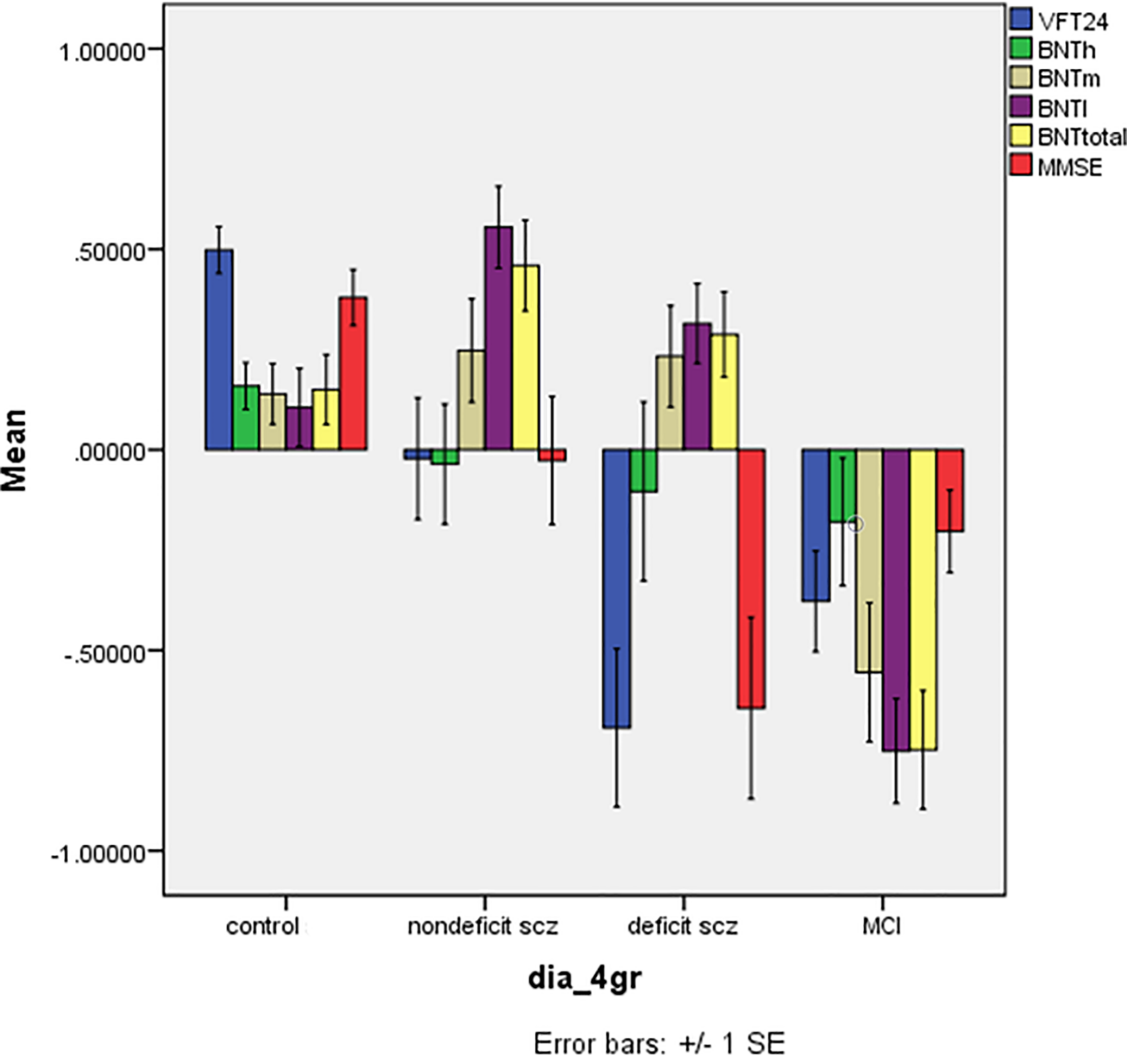
Mean (SE) z scores of Consortium to Establish a Registry for Alzheimer’s disease tests in normal controls, nondeficit and deficit schizophrenia patients and patients with amnestic mild cognitive impairment (MCI). VFT24: verbal fluency test; BNT: Modified Boston Naming Test; BNTh: naming of highly frequency objects; BNTm: naming of medium frequency objects; BNTl: naming of low frequency objects; BNTtotal: BNT total score. MMSE: Mini Mental State Examination.

**Fig 1** shows the differences in the z values of the VFT, BNT and MMSE test scores in the 4 study groups. There was a highly significant effect of diagnostic groups (F = 10.18, df = 15/646, p<0.001) explaining 17.7% of the variance in the data. Tests for between-subject effects showed significant effects on VFT (F = 22.65, df = 3/238, p<0.001, partial eta-squared = 0.222), BNTm (F = 9.20, df = 3/238, p<0.001, partial eta-squared = 0.104), BNTl (F = 21.29, df = 3/238, p<0.001, partial eta-squared = 0.212), BNT total (F = 19.44, df = 3/238, p<0.001, partial eta-squared = 0.197), and MMSE (F = 22.65, df = 3/238, p<0.001, partial eta-squared = 0.222). Post-hoc analyses showed lower VFT values in aMCI and deficit patients as compared with controls and nondeficit patients. There was a trend towards lowered VFT scores in patients with deficit schizophrenia as compared with aMCI patients (p = 0.082). Total BNT scores were significantly lower in aMCI than in the other three groups, which did not differ significantly. The MMSE was significantly lower in deficit schizophrenia than in the other three groups, whilst MMSE was also lower in aMCI than in controls, but not nondeficit schizophrenia patients.

Table 3 First row, shows the model-generated estimated marginal means of the z-values obtained by multivariate GLM analyses after adjustment for age, education and sex. Diagnosis had a significant effects on the 6 CERAD measurements (F = 5.66, df = 15/638, p<0.001, partial eta-squared = 0.109), while also age (F = 5.01, df = 5/231, p<0.001, partial eta-squared = 0.098), education (F = 23.56, df = 3/231, p<0.001, partial eta-squared = 0.338), and gender (F = 4.77, df = 5/231, p<0.001, partial eta-squared = 0.094) had significant effects. Table 3 shows the differences between the 4 study groups in the 6 variables and the results of post-hoc analyses. Thus, the VFT was lower in all patient groups compared with controls, and lower in deficit schizophrenia as compared with all other study groups, while there were no differences between aMCI and nondeficit schizophrenia.

### WLM score differences between the diagnostic groups

**Fig 2:**
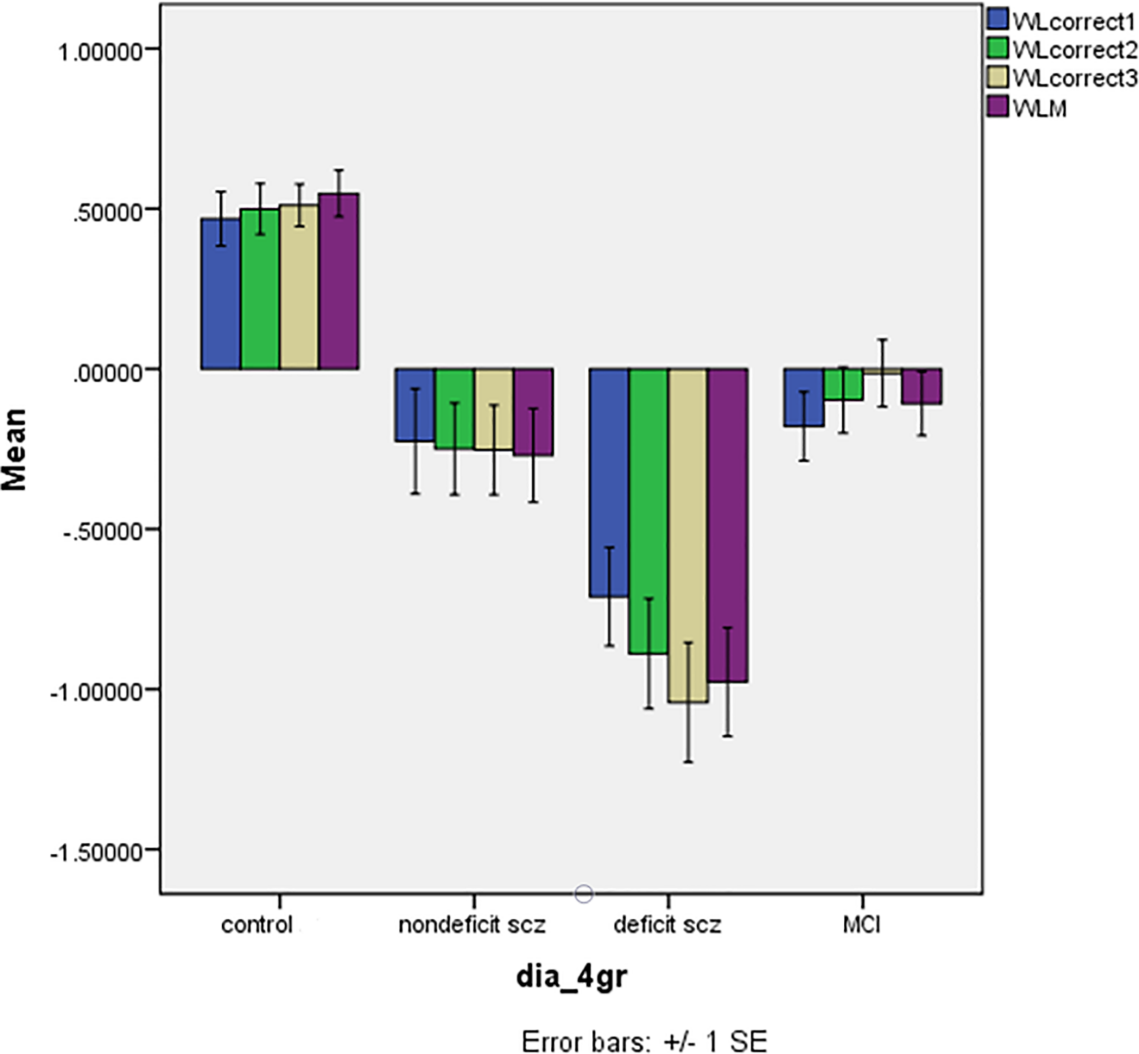
Mean (SE) z scores of Consortium to Establish a Registry for Alzheimer’s disease tests in normal controls, nondeficit and deficit schizophrenia patients and patients with amnestic mild cognitive impairment (MCI). WLcorrect1: Trial 1 (WLM correct 1); WLcorrect2: Trial 2 (WLM correct 2); WLcorrect3: Trial 3 (WLM correct 3); WLM: Word List Memory, total score.

**Fig 2** shows the differences in the z values of the WLM correct1, correct2, correct3 and WLM total in the 4 study groups. There was a highly significant effect of diagnostic groups (F = 11.41, df = 9/577, p<0.001) explaining 12.4% of the variance in the data. Tests for between-subject effects showed significant effects on WLM correct1 (F = 19.11, df = 3/239, p<0.001, partial eta-squared = 0.193), WLM correct2 (F = 26.47, df = 3/239, p<0.001, partial eta-squared = 0.249), WLM correct3 (F = 34.28, df = 3/239, p<0.001, partial eta-squared = 0.301), and WLM total (F = 34.20, df = 3/239, p<0.001, partial eta-squared = 0.300). The 4 WLM variables were significantly lower in deficit schizophrenia than in the three other groups and lower in aMCI than in controls, while there were no differences between aMCI and nondeficit schizophrenia.

Table 3 (row 2) shows the differences between the 4 study groups in the 4 WLM variables and the results of post-hoc analyses. Thus, there were highly significant differences between the 4 groups in all 4 variables. WLM correct1, WLM correct2 and WLM total were significantly lower in all three patient groups as compared with controls, lower in deficit schizophrenia than in the three other study groups, and lower values in nondeficit schizophrenia than in aMCI. There was a significant effect of gender (F = 4.70, df = 3/234, p = 0.003; partial eta-squared = 0.057) and education (F = 8.56, df = 3/234, p<0.001; partial eta-squared = 0.099). The four WLM scores were significantly higher in females than males (all p<0.001). Education was significantly and positively associated with all 4 scores (all p<0.004).

### Word List Recall and recognition differences between the diagnostic groups

**Fig 3:**
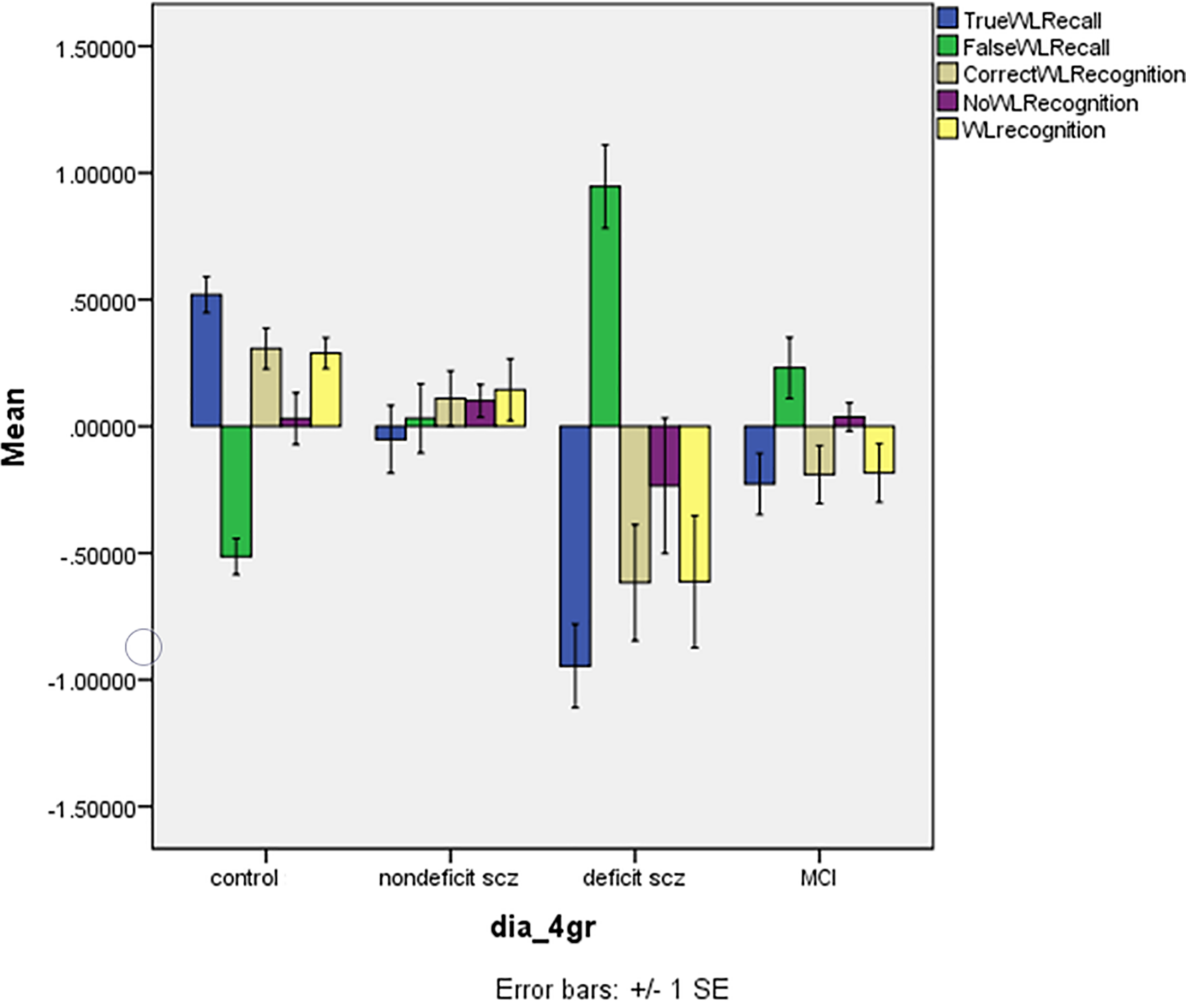
Mean (SE) z scores of Consortium to Establish a Registry for Alzheimer’s disease tests in normal controls, nondeficit and deficit schizophrenia patients and patients with amnestic mild cognitive impairment (MCI). TrueWLRecall: Word List Recall, Delayed, true recall; FalseWLRecall: Word List Recall, Delayed, false recall (WL False Recall); CorrectWLRecognition: WL Recognition Correct Yes response; NoWLRecognition: Word List Recognition Correct No response; WLRecognition: sum of Correctand No WLRecognition.

**Fig 3** shows the differences in the z values of the WL Recall and Recognition scores in the 4 study groups. There was a highly significant effect of diagnostic groups (F = 6.17, df = 15/649, p<0.001) explaining 11.5% of the variance in the data. Tests for between-subject effects showed significant effects on WL True Recall (F = 30.34, df = 3/239, p<0.001, partial eta-squared = 0.276), WL False Recall (F = 30.14, df = 3/239, p<0.001, partial eta-squared = 0.274), WLRecCorrect (F = 10.26, df = 3/239, p<0.001, partial eta-squared = 0.114), and WLRec total (F = 9.79, df = 3/239, p<0.001, partial eta-squared = 0.109). Post-hoc analyses shows that WL True Recall was significantly lower in deficit schizophrenia than in the three other groups and lower in aMCI and nondeficit schizophrenia than in controls. WL False Recall was significantly higher in deficit schizophrenia than in the three other groups and higher in aMCI and nondeficit schizophrenia than in controls. WLRecCorrect and WLRec total were significantly lower in deficit schizophrenia than in the three other groups and lower in aMCI, but not nondeficit schizophrenia, than in controls.

Table 3 shows the differences between the 4 study groups in the 5 WL recall and recognition variables and the results of multivariate GLM and post-hoc analyses. There was also a significant effect of education (F = 5.09, df = 5/232, p<0.001) but not age or sex. WL True Recall was significantly lower in the three patients groups as compared with controls, lower in deficit schizophrenia as compared with the three other groups but no differences between nondeficit schizophrenia and aMCI. WL False Recall was significantly higher in the three patients groups as compared with controls and higher in deficit schizophrenia as compared with the three other groups, whilst there were no significant differences between nondeficit schizophrenia and aMCI. WLRecCorrect and WLRec total were significantly lower in deficit schizophrenia than in the three other groups. Parameter estimates show that all variables (except WLRecNoCorrect) were inversely associated with education.

### Results of binary logistic regression analysis

Table 4 shows the results of binary logistic regression analysis with aMCI and (non)deficit schizophrenia as dependent variables and the CERAD tests as explanatory variables. We found that both BNT and WLM were associated with aMCI versus deficit schizophrenia with a large effect size. Lower BNT and increased WLM are strongly associated with aMCI versus deficit schizophrenia. The same variables together with lowered WL Recognition were also associated with aMCI versus nondeficit schizophrenia.

**Table 4:** Results of binary logistic regression analyses with amnestic mild cognitive impairment (aMCI) versus deficit or non-deficit schizophrenia as classes and 7 Consortium to Establish a Registry for Alzheimer’s disease (CERAD) tests as explanatory variables.

Entered as explanatory variables	Nagelkerke	Significant explanatory variables	Wald	df	P	OR	95% CI
**aMCI versus deficit**	0.536	BNT	19.66	1	<0.001	0.15	0.06–0.34
	50.54, df = 2, p<0.001	WLM	14.66	1	<0.001	7.72	2.13–10.45
**aMCI versus non-deficit**	0.454	BNT	20.13	1	<0.001	0.23	0.12–0.44
	40.44, df = 2,	WLM	7.41	1	0.006	2.67	1.32–5.43
	p<0.001	WLRec total	4.12	1	0.042	0.42	0.19–0.97

OR: Odds ratio, 95% CI: confidence intervals BNT: total scores on the Boston Naming Test. WLM: total scores on the Word List Memory test. WLRec total: total scores on the Word List Recognition test.

### Results of Linear SVM and Random Forest Models

Table 5 shows the results of 10-fold cross-validation and predictive weights or importances of the CERAD variables in Linear SVM and Random Forest Models separating aMCI from deficit or nondeficit schizophrenia. We trained two different models, a first with the CERAD scores and a second with CERAD scores and age, sex and education. Indeed, given the significant associations between age and BNT and MMSE variables (see Table 2) and education and sex (see results of GLM analyses) we also examined whether using CERAD features provide any predictive power in the presence of these confounding variables. Using the CERAD features to segregate aMCI from deficit schizophrenia resulted in a good 10-fold cross-validation accuracy for Linear SVM as well as Random Forest. Both types of analyses (with and without the confounders) showed that BNT and WLM belonged to the top-3 variables in all models. When examining the 10-fold cross-validation accuracy in aMCI versus nondeficit schizophrenia we found a somewhat less adequate accuracy of the CERAD features. The two types of procedures showed that BNT was the most predictive feature while WLM was consistently among the top 3 features in three out of four models. Overall, binary logistic regression analyses, Linear SVM and Random Forest (including after adjustment for the confounders were made) agreed that BNT and WLM are the key features segregating aMCI from deficit and nondeficit schizophrenia. S1 Fig and S2 Fig show the Random Forest predicted class probabilities and Linear SVM distances to decision boundary for aMCI versus deficit and nondeficit schizophrenia, respectively.

**Table 5:** Results of Linear SVM and Random Forest Models.

**aMCI versus Deficit SCZ**	**Linear SVM**			**Random Forest**		
	**Validation accuracy (%)**	**Top-features**	**Feature weights**	**Validation accuracy (%)**	**Top-features**	**Feature importances**
7 CERAD	81.0 (13.0)	BNT	-0.6682	78.0 (10.7)	BNT	0.2991
		WL	-0.3866		WLM	0.2489
		False Recall	0.2759		VFT	0.1390
		WLM				
7 CERAD	97.0 (4.6)	BNT	-0.2699	98.0 (4.0)	BNT	0.1977
- Age		WLM	0.2606		WLM	0.0925
- Sex		VFT	0.2465		WL	0.0406
- Education					False Recall	
**aMCI versus Nondeficit SCZ**	**Validation accuracy (%)**	**Top-features**	**Feature weights**	**Validation accuracy (%)**	**Top-features**	**Feature importances**
CERAD	73.6 (10.9)	BNT	-0.5626	71.4 (14.3)	BNT	0.3410
		WLM	0.3562		WLM	0.2226
		WLRec total	-0.2955		VFT	0.1882
7 CERAD,	100.0 (0.0)	BNT	-0.3144	98.9 (3.3)	BNT	0.1545
- Age		WL	0.3013		WL	0.0573
- Sex		False Recall			False Recall	
- Education		WLM	0.2719		VFT	0.0477

10-fold cross-validation performances and predictive weights in Support Vector Machine with linear kernel (Linear SVM) and importances in Random Forest Models performed using 7 Consortium to Establish a Registry for Alzheimer’s disease (CERAD) test results as explanatory variables and amnestic mild cognitive impairment (aMCI) versus (non)deficit schizophrenia (SCZ) as dichotomous classes. VFT: Verbal Fluency Test. BNT: Boston Naming Test. WLM: Word List Memory. WL False Recall: Word List Recall, Delayed, false recall. WLRec total: Word List Recognition total.

### Neural networks

Table 6 shows the results of MLP Neural networks predicting aMCI versus deficit schizophrenia (output or target variables) using the CERAD scores as input variables. Automatic architecture training of the MLP neural network delineated the best model with 2 hidden layers with 4 units in layer 1 and 3 units in layer 2, and with hyperbolic tangent as activation function in layer 1 and softmax in hidden layer 2. The differences in cross-entropy error between the training and testing sets show that the neural model has learnt to generalize from the trend. The rate of incorrect predictions was 20.0% in the training sample, 13.3% in the testing sample and 20.0% in the holdout sample. The partitioned confusion matrices (Table 6) show that in the training sample 85.2% of the aMCI patients were correctly classified and 72.2% of the deficit patients. The accuracy of the holdout sample showed 91.7% correctly classified aMCI patients and 62.5% deficit patients, whilst the AUC ROC curve was 0.931.

**Table 6:** Results of Multilayer Perceptron Neural Network analysis with 7 Consortium to Establish a Registry for Alzheimer’s disease (CERAD) tests.

**aMCI versus Deficit**	**Samples**	**Cross entropy error term**	**% incorrect predictions**	**Observed**	**Predicted**	**AUC ROC**
7 CERAD tests	Training	16.488	20.0%	Deficit	72.2%	0.931
				MCI	85.2%	
	Testing	5.288	13.3%	Deficit	83.3%	
				MCI	88.9%	
	Holdout	-	20.0%	Deficit	62.5%	
				MCI	91.7%	
**aMCI versus NON-deficit features**	**Samples**	**Sum of squares error term**	**% incorrect predictions**	**Observed**	**Predicted**	**AUC ROC**
7 CERAD tests	Training	6.176	23.9%	Non-deficit	78.9%	0.898
				MCI	74.1%	
	Testing	2.246	18.2%	Non-deficit	75.0%	
				MCI	85.7%	
	Holdout	-	25.8%	Non-deficit	50.0%	
				MCI	89.5%	

Results as input variables and amnestic mild cognitive impairment (aMCI) versus (non)deficit schizophrenia as dichotomous output variables. Shown are the error terms, percentage incorrect predictions, the partitioned confusion matrix (predicted) and the area under the ROC curve (AUC ROC)

**Fig 4:**
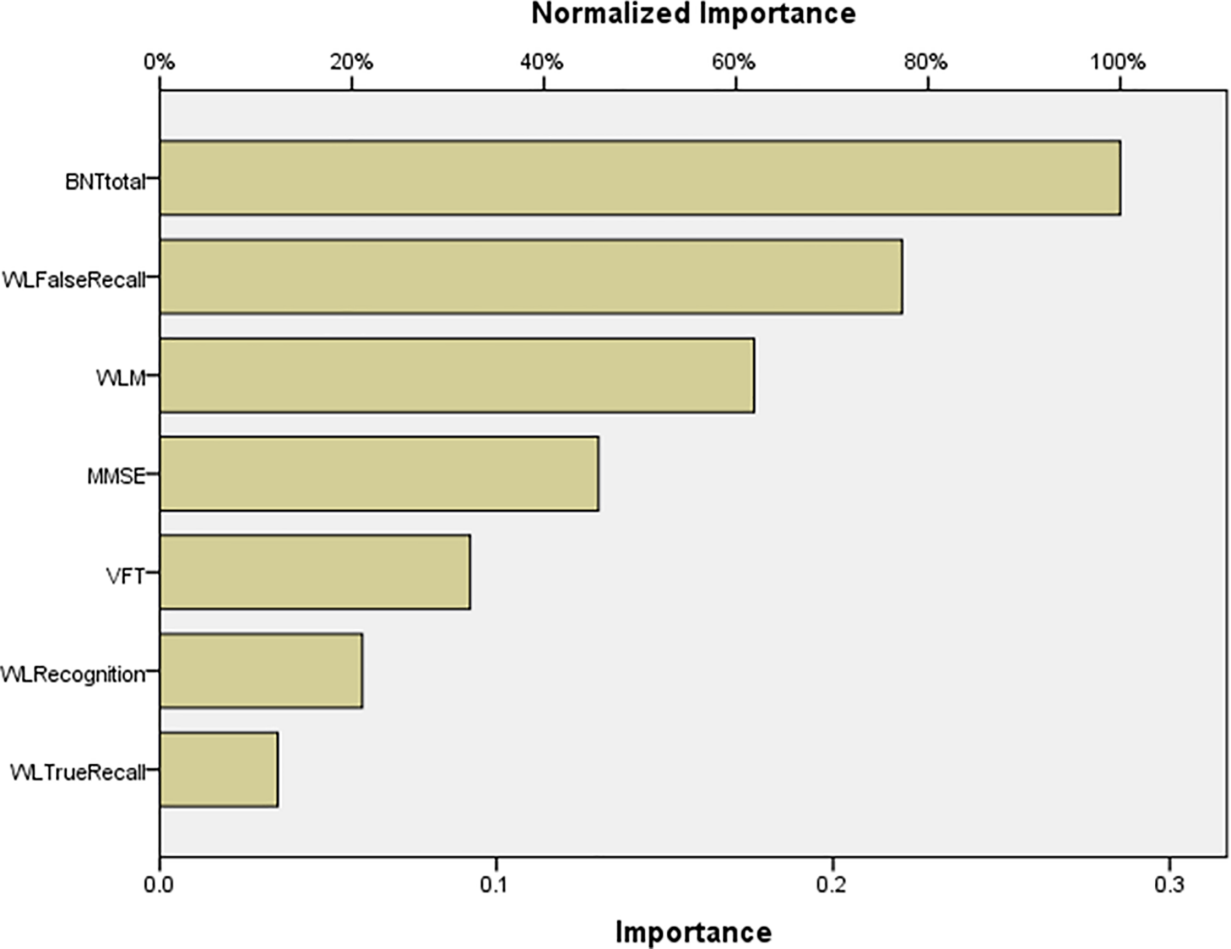
Neural network importance chart showing the relative and normalized importances of the Consortium to Establish a Registry for Alzheimer’s disease test scores as input variables predicting amnestic mild cognitive impairment versus deficit schizophrenia (output variables). BNTtotal: Boston naming Test, total score; WLM: Word List Memory; WLFalse Recall: Word List Recall, Delayed, false recall; MMSE: Mini Mental State Examination; WLTRueRecall: Word List Recall, Delayed, true recall; VFT: Verbal Fluency Test; WLRecognition: Word List Recognition, total score.

**Fig 4** shows the impact of the input variables in the neural model as relative and normalized importances. BNT total was the most important determinant of the predictive power of the neural model, followed at a distance by WL False Recall, WLM and MMSE. S3 Fig shows the results of this Multilayer Perceptron Neural Network analysis with network information, model summary, parameter estimates, and input variable importances.

Table 6 also shows the results of MLP Neural networks predicting aMCI and nondeficit schizophrenia using the CERAD scores as input variables. Automatic architecture training identified the best model with 2 hidden layers with 3 and 2 units in layer 1 and 2, respectively, and with hyperbolic tangent as activation function in layer 1 and identity in hidden layer 2. In the training sample, the rate of incorrect predictions was 23.9% and this was lower in the testing (18.2%) sample, while the holdout sample showed 25.8% incorrect predictions. The overall confusion matrix showed that 76.1% of all cases were correctly classified in the training sample, while this increased to 81.8% in the testing sample. The AUC under the ROC curve was 0.898.

**Fig 5:**
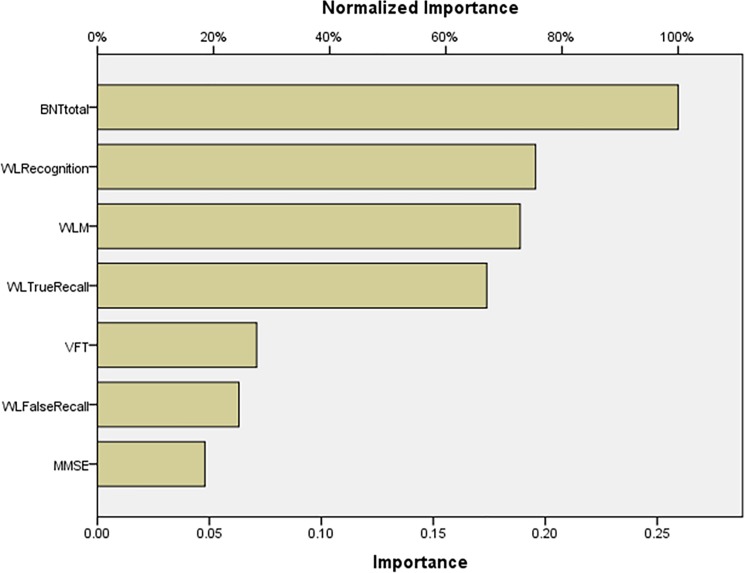
Neural network importance chart showing the relative and normalized importances of the Consortium to Establish a Registry for Alzheimer’s disease test scores as input variables predicting amnestic mild cognitive impairment versus nondeficit schizophrenia (output variables). BNTtotal: Boston naming Test, total score; WLM: Word List Memory; WLTRueRecall: Word List Recall, Delayed, true recall; WLRecognition: Word List Recognition, total score; WLFalse Recall: Word List Recall, Delayed, false recall; VFT: Verbal Fluency Test; MMSE: Mini Mental State Examination.

**Fig 5** shows the relative and normalized importances of the CERAD data contributing to the predictive power of the model. BNT total was the dominant determinant, while WL Recognition, WLM and WL True Recall followed at a distance. S4 Fig .pdf shows the results of this Multilayer Perceptron Neural Network analysis with network information, model summary, parameter estimates, and input variable importances.

## Discussion

The main finding of this study is that deficit schizophrenia and aMCI show major differences in neurocognitive profiles. Results of GLM and logistic regression analyses, linear SVM, Random Forest and Neural Networks agreed that deficit schizophrenia is well-separated from aMCI patients by lowered WLM and MMSE test scores and higher false recall scores, while patients with aMCI show lower BNT scores. Moreover, aMCI patients were significantly separated from controls by lower VFT, BNT, MMSE, and WLM test results. These results show that impairments in episodic memory, immediate working memory and recall are characteristic features of deficit schizophrenia, whilst dysnomia is a feature of aMCI. It is interesting to note that after considering the effects of BNT in logistic regression analyses, SVM and Random Forest and Neural Networks, WLM was also a significant feature of nondeficit schizophrenia versus aMCI.

Different studies, reviews and meta-analyses show deficits in episodic or working memory in patients with schizophrenia [28–32]. Another recent meta-analysis shows that both types of schizophrenia are accompanied by impairments in general cognitive capacities, visuospatial memory and sustained attention, while these deficits are generally more pronounced in deficit than in nondeficit schizophrenia [33]. Previous reports show that semantic memory tests and immediate recall are impaired in MCI as compared with controls [10]. In other studies, WLM and WL Recognition tests distinguished MCI patients from healthy controls [11,13]. Language deficits including poor naming performance are early and important diagnostic criteria for MCI [16,34,35]. Overall, the cognitive profile of MCI patients (versus controls) shows more impairments in naming, verbal fluency, verbal memory and recall as compared with controls.

In our study, WLM and WL True Recall scores in younger patients with deficit schizophrenia were 0.869 and 0.745 standard deviations below the mean of elderly aMCI subjects, while naming scores were 1.035 standard deviations lower in aMCI than deficit schizophrenia patients. Therefore, the correct interpretation of the current results is that younger patients with deficit schizophrenia have more severe impairments in episodic memory, delayed recall and recognition than elderly patients with MCI, and that the latter have more dysnomia than younger patients with deficit schizophrenia. Thus, while memory disturbances are considered to be the main features of aMCI [6], episodic memory tests are even more impaired in younger subjects with deficit schizophrenia than in elderly subjects with aMCI. Interestingly, in our Random Forest models and age-adjusted regression analyses, lowered VFT scores were found in deficit schizophrenia versus aMCI patients. Thus, while low VFT performance is a characteristic of MCI [36,37], verbal fluency was even lower in deficit schizophrenia than in aMCI. These findings extend the knowledge that patients with schizophrenia, and especially those with deficit schizophrenia, show reduced independence, poor social functioning, ADL impairments and worse physical health than normal controls [38–40]. Although patients with MCI make more errors performing complex tasks, including shopping and paying bills, a decreased independence, as detected in deficit schizophrenia, is not a feature of MCI [6].

The highly significant differences in cognitive profiles between both (deficit) schizophrenia and aMCI and the significantly greater memory disturbances in younger schizophrenia subjects than in elderly aMCI participants indicate that different pathways underpin both conditions. There is a vast literature on neurobiological mechanisms underlying the neuropsychological dysfunction in schizophrenia supporting neurodevelopmental origins of cognitive decline in schizophrenia [41]. Thus, schizophrenia is considered to be a neuroprogressive disorder caused by multiple hits causing neuronal disruption, epigenetic changes, and activation of immune-inflammatory and oxidative stress pathways which further impair neuronal functions [41]. More specifically, the biosignature of memory impairments in (deficit) schizophrenia is highly significantly associated with activation of the tryptophan catabolite (TRYCAT) pathway and increased activity of neurotoxic TRYCATs, including picolinic and xanthurenic acid [18]. These associations may be ascribed to the antecedents (including immune activation or neuro-oxidative stress) or consequents (including increased neuroprogression) of TRYCAT pathway activation [18]. Interestingly, a recent review shows that the amyloid cascade is not associated with neurocognitive impairments in schizophrenia [41]. In MCI, however, low memory and MMSE scores are significantly associated with amyloid positivity [42,43], while increased cerebro-spinal fluid amyloid β42 and t-Tau are the best predictors of conversion from MCI to AD [43]. Moreover, impairments in episodic memory in schizophrenia may be due to the lack of awareness [44], while cognitive impairments in MCI may represent deficits that may be associated with incipient dementia [45]. In our study, increasing age is accompanied by lowered VFT, BNT and MMSE scores, which are also the key features of aMCI, suggesting that accelerated ageing in processes that mediate language and executive-semantic functions play an important role in aMCI. One theory is that age-related changes in central pathways make the brain more vulnerable to neurodegenerative processes thereby accelerating detrimental effects of ageing [46].

Finally, lower education could be another characteristic of aMCI and deficit schizophrenia. In our normal controls, years of education was positively associated with BNT, MMSE, WLM and WL true recall and recognition test scores and negatively with WL false recall scores. This suggests that higher education could constitute a protective factor against the cognitive decline occurring in patients with aMCI and (non)deficit schizophrenia. Previously, it was shown that education was protective for MCI [47]. Moreover, it is known that education may contribute to cognitive reserve, which enhances resilience to brain damage by fostering more adequate cognitive strategies and a better exploitation of intact brain networks [48–50].

A limitation of this study is its cross-sectional design, which precludes the establishment of firm causal inferences. Future research should examine other age-associated cognitive domains, which may contribute to a better differentiation between aMCI and deficit schizophrenia, including visuospatial skills, executive functions, paired association learning and spatial working memory. It could be argued that the Measurement and Treatment Research to Improve Cognition in Schizophrenia, Consensus Cognitive Battery (MATRICS) would be a more adequate choice to assess the different cognitive profiles in patients with schizophrenia [51]. Nevertheless, to the best of our knowledge no previous studies have shown the validity of the MATRICS in aMCI, while the CERAD is useful to assess episodic and semantic memory impairments (and to a lesser degree also executive functions) in aMCI [10–16] and schizophrenia [18]. In addition, there is some evidence that impairments in episodic and semantic memory are more strongly associated with deficit schizophrenia than executive functions [52]. Finally, MCI comprises three subtypes, namely aMCI (examined in our study) characterized by deficits in memory, multiple-domain MCI characterized by multiple neurocognitive impairments, and single non-amnestic MCI characterized by deficits in other domains [53]. Therefore, it is possible that the two latter MCI subtypes may show other cognitive profiles. The main strengths of the current study rest on the use of a well-established neuropsychological test battery to measure cognitive decline in well characterized study samples. Another strength is that we employed machine learning procedures, including neural networks, which have the ability to define complex predictive models for dichotomous outcome variables [54].

## Conclusions

Regression analyses and machine learning procedures show that schizophrenia, and especially deficit schizophrenia, is best separated from aMCI using BNT and WLM scores as explanatory variables. The impairments in episodic memory, recall and recognition are more severe in younger patients with deficit schizophrenia as compared with elderly patients with aMCI, while aMCI patients show more dysnomia than patients with schizophrenia. These findings indicate that impaired episodic memory is a feature of (non)deficit schizophrenia, whilst dysnomia is a feature of aMCI.

## Supporting information

S1 Fig Forest and Linear Support Vector Machine (Linear SVM) models. Random Forest predicted class probabilities and Linear SVM distances to decision boundary for mild cognitive impairment (MCI) versus deficit schizophrenia. Lower scores (probabilities or distances) are associated with MCI.
(PDF)

S2 Fig Forest and Linear Support Vector Machine (Linear SVM) models. Random Forest predicted class probabilities and Linear SVM distances to decision boundary for mild cognitive impairment (MCI) versus nondeficit schizophrenia (SCZ). Lower scores (probabilities or distances) are associated with MCI.
(PDF)

S3 Fig Neural Network analysis: Deficit schizophrenia versus aMCI. Shown are the results of Multilayer Perceptron Neural Network analysis with network information, model summary, parameter estimates, and input variable importance. Area under the curve and classification results are shown in Table 6.
(PDF)

S4 Fig Neural Network analysis: Separation non-deficit schizophrenia versus aMCI. Shown are the results of Multilayer Perceptron Neural Network analysis with network information, model summary, parameter estimates, and input variable importance. Area under the curve and classification results are shown in Table 6.
(PDF)
