# Supplementary material for: Episodic memory and delayed recall are significantly more impaired in younger patients with deficit schizophrenia than in elderly patients with amnestic mild cognitive impairment
Source: PLoS One. 2018 May 15;13(5):e0197004. doi: 10.1371/journal.pone.0197004 (PMC5953437; doi:10.1371/journal.pone.0197004)
Supplement: S1 Fig — Random Forest predicted class probabilities and Linear SVM distances to decision boundary for mild cognitive impairment (MCI) versus deficit schizophrenia. Lower scores (probabilities or distances) are associated with MCI. (PDF) [file pone.0197004.s001.pdf]

Episodic memory and delayed recall are significantly more impaired in younger patients with deficit schizophrenia than in elderly patients with amnestic mild cognitive impairment.

Short title: deficit schizophrenia and mild cognitive impairment

Buranee Kanchanatawan, Sookjaroen Tangwongchai, Thitiporn Supasitthumrong, Sira Sriswasdi, Michael Maes

S1_File.pdf. Forest and Linear Support Vector Machine (Linear SVM) models

Random Forest predicted class probabilities and Linear SVM distances to decision boundary for mild cognitive impairment (MCI) versus deficit schizophrenia (SCZ). Lower scores (probabilities or distances) are associated with MCI.

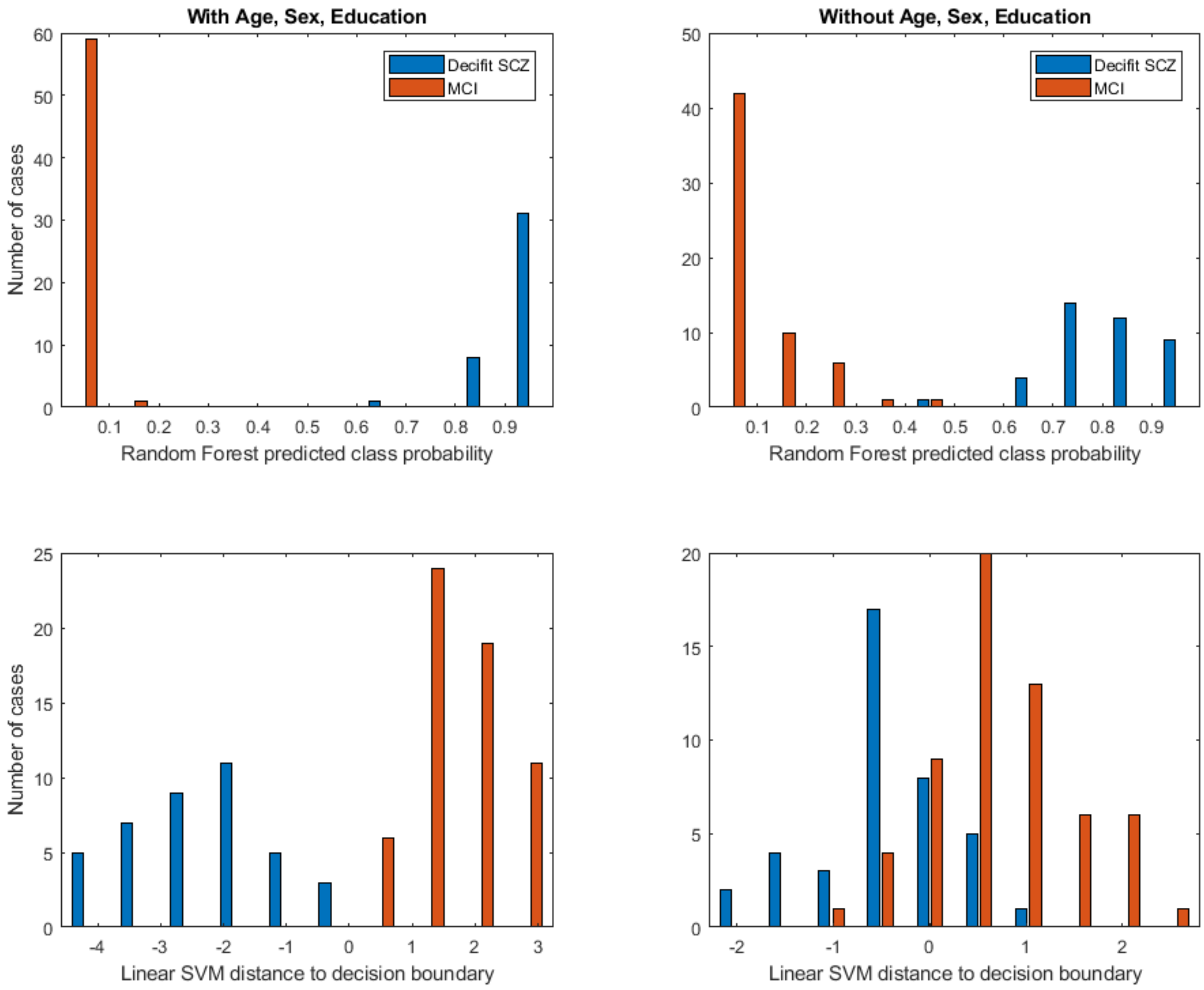

With Age, Sex, Education
Without Age, Sex, Education
Decifit SCZ
MCI
Number of cases
Random Forest predicted class probability
Linear SVM distance to decision boundary
