# Supplementary material for: Episodic memory and delayed recall are significantly more impaired in younger patients with deficit schizophrenia than in elderly patients with amnestic mild cognitive impairment
Source: PLoS One. 2018 May 15;13(5):e0197004. doi: 10.1371/journal.pone.0197004 (PMC5953437; doi:10.1371/journal.pone.0197004)
Supplement: S3 Fig — Shown are the results of Multilayer Perceptron Neural Network analysis with network information, model summary, parameter estimates, and input variable importance. Area under the curve and classification results are shown in Table 6. (PDF) [file pone.0197004.s003.pdf]

Episodic memory and delayed recall are significantly more impaired in younger patients with deficit schizophrenia than in elderly patients with amnestic mild cognitive impairment.

Short title: deficit schizophrenia and mild cognitive impairment

Buranee Kanchanatawan,* Sookjaroen Tangwongchai, * Thitiporn Supasitthumrong, Sira Sriswasdi, Michael Maes

* shared first authorship

Neural Network Analysis: Deficit schizophrenia versus aMCI

Shown are the results of Multilayer Perceptron Neural Network analysis with network information, model summary, parameter estimates, and input variable importance.

Network Information

Input Layer	Covariates	1	VFT
		2	BNTtotal
		3	MMSE
		4	WLM

		5	WL True Recall
		6	WL False Recall
		7	WLRecognition
	Number of Units[a]		7
	Rescaling Method for Covariates		Standardized
Hidden Layer(s)	Number of Hidden Layers		2
	Number of Units in Hidden Layer 1[a]		4
	Number of Units in Hidden Layer 2[a]		3
	Activation Function		Hyperbolic tangent
Output Layer	Dependent Variables	1	deficit_MCI
	Number of Units		2
	Activation Function		Softmax
	Error Function		Cross-entropy

a. Excluding the bias unit

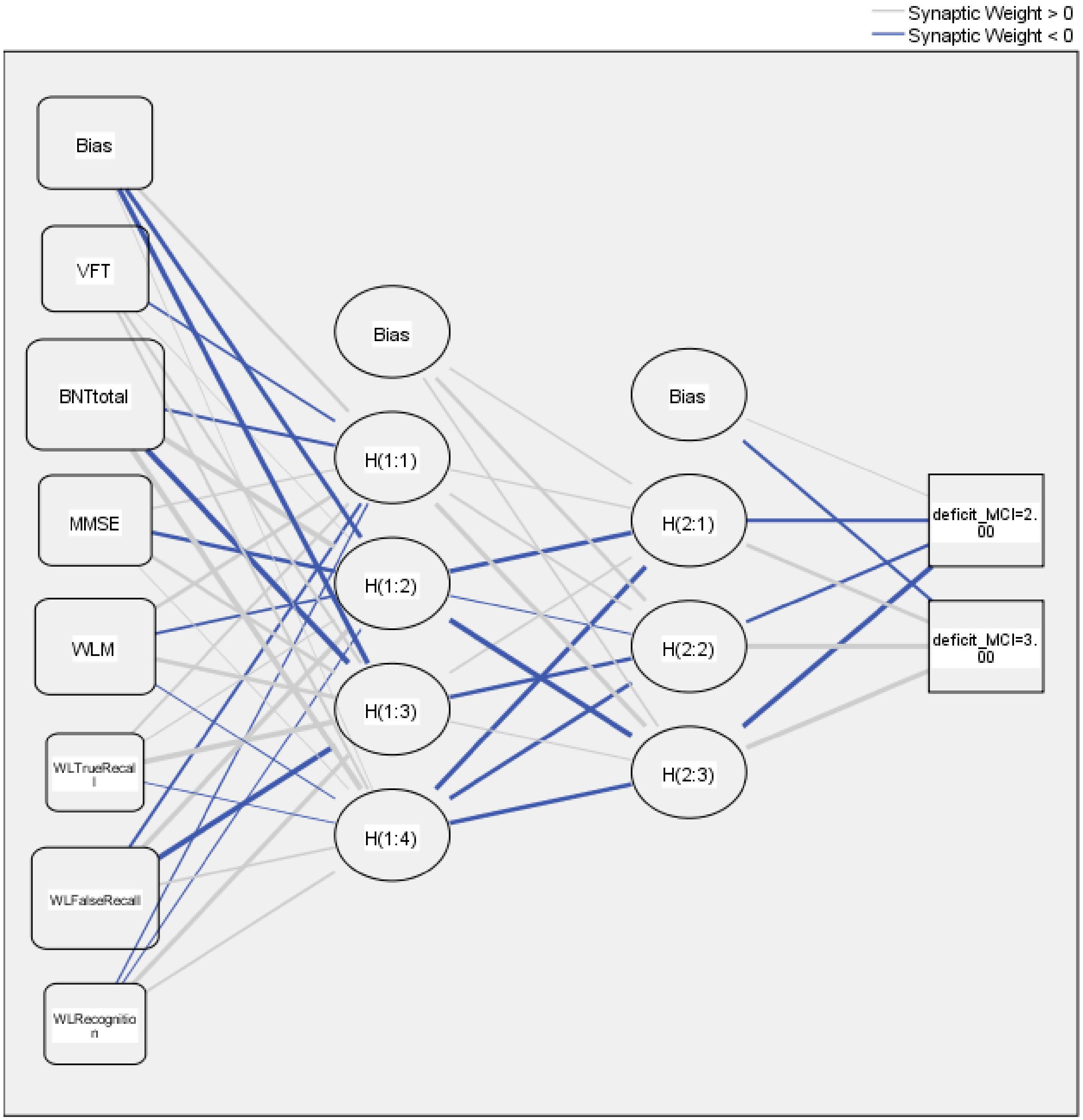

Hidden layer activation function: Hyperbolic tangent

Output layer activation function: Softmax

Model Summary

Training	Cross Entropy Error	16.488
	Percent Incorrect Predictions	20.0%
	Stopping Rule Used	1 consecutive step(s) with no decrease in error[a]
	Training Time	0:00:00.01
Testing	Cross Entropy Error	5.288
	Percent Incorrect Predictions	13.3%
Holdout	Percent Incorrect Predictions	20.0%

Dependent Variable: deficit_MCI

a. Error computations are based on the testing sample.

Parameter Estimates

Predictor		Predicted								
		Hidden Layer 1				Hidden Layer 2			Output Layer	
		H(1:1)	H(1:2)	H(1:3)	H(1:4)	H(2:1)	H(2:2)	H(2:3)	[deficit_MCI=2.00]	[deficit_MCI=3.00]
Input Layer	(Bias)	.408	-.612	-.720	.055					
	VFT	-.256	.047	.363	.315					
	BNTtotal	-.371	1.007	-1.239	1.308					
	MMSE	.196	-.471	.431	.072					
	WLM	.436	-.298	.548	-.101					
	WLTrueRecall	.309	.163	1.105	-.074					
	WLFalseRecall	-.346	.671	-.933	.258					
	WLRecognition	-.183	-.153	.614	.286					
Hidden Layer 1	(Bias)					.219	.435	.229		
	H(1:1)					.189	.258	.646		
	H(1:2)					-.620	-.144	-.795		
	H(1:3)					.241	-.449	.176		
	H(1:4)					-.650	-.509	-.555		
Hidden Layer 2	(Bias)								.100	-.367
	H(2:1)								-.398	.440
	H(2:2)								-.378	.780
	H(2:3)								-.734	.679

Independent Variable Importance

	Importance	Normalized Importance
VFT	.092	32.3%
BNTtotal	.285	100.0%
MMSE	.130	45.7%
WLM	.177	61.9%
WL True Recall	.035	12.3%
WL False Recall	.220	77.3%
WLRecognition	.060	21.1%
