# Supplementary material for: Episodic memory and delayed recall are significantly more impaired in younger patients with deficit schizophrenia than in elderly patients with amnestic mild cognitive impairment
Source: PLoS One. 2018 May 15;13(5):e0197004. doi: 10.1371/journal.pone.0197004 (PMC5953437; doi:10.1371/journal.pone.0197004)
Supplement: S4 Fig — Shown are the results of Multilayer Perceptron Neural Network analysis with network information, model summary, parameter estimates, and input variable importance. Area under the curve and classification results are shown in Table 6. (PDF) [file pone.0197004.s004.pdf]

Episodic memory and delayed recall are significantly more impaired in younger patients with deficit schizophrenia than in elderly patients with amnestic mild cognitive impairment.

Short title: deficit schizophrenia and mild cognitive impairment

Buranee Kanchanatawan,* Sookjaroen Tangwongchai, * Thitiporn Supasitthumrong, Sira Sriswasdi, Michael Maes

* shared first authorship

Supplementary Material

Neural Network Analaysis: Separation Non-deficit schizophrenia and aMCI

Shown are the results of Multilayer Perceptron Neural Network analysis with network information, model summary, parameter estimates, and input variable importance.

Network Information

Input Layer	Covariates	1	VFT
		2	BNTtotal
		3	MMSE
		4	WLM
		5	WL True Recall
		6	WL False Recall
		7	WLRecognition
	Number of Units[a]		7
	Rescaling Method for Covariates		Standardized
Hidden Layer(s)	Number of Hidden Layers		2
	Number of Units in Hidden Layer 1[a]		3
	Number of Units in Hidden Layer 2[a]		2
	Activation Function		Hyperbolic tangent
Output Layer	Dependent Variables	1	nondeficit_mci
	Number of Units		2
	Activation Function		Identity
	Error Function		Sum of Squares

a. Excluding the bias unit

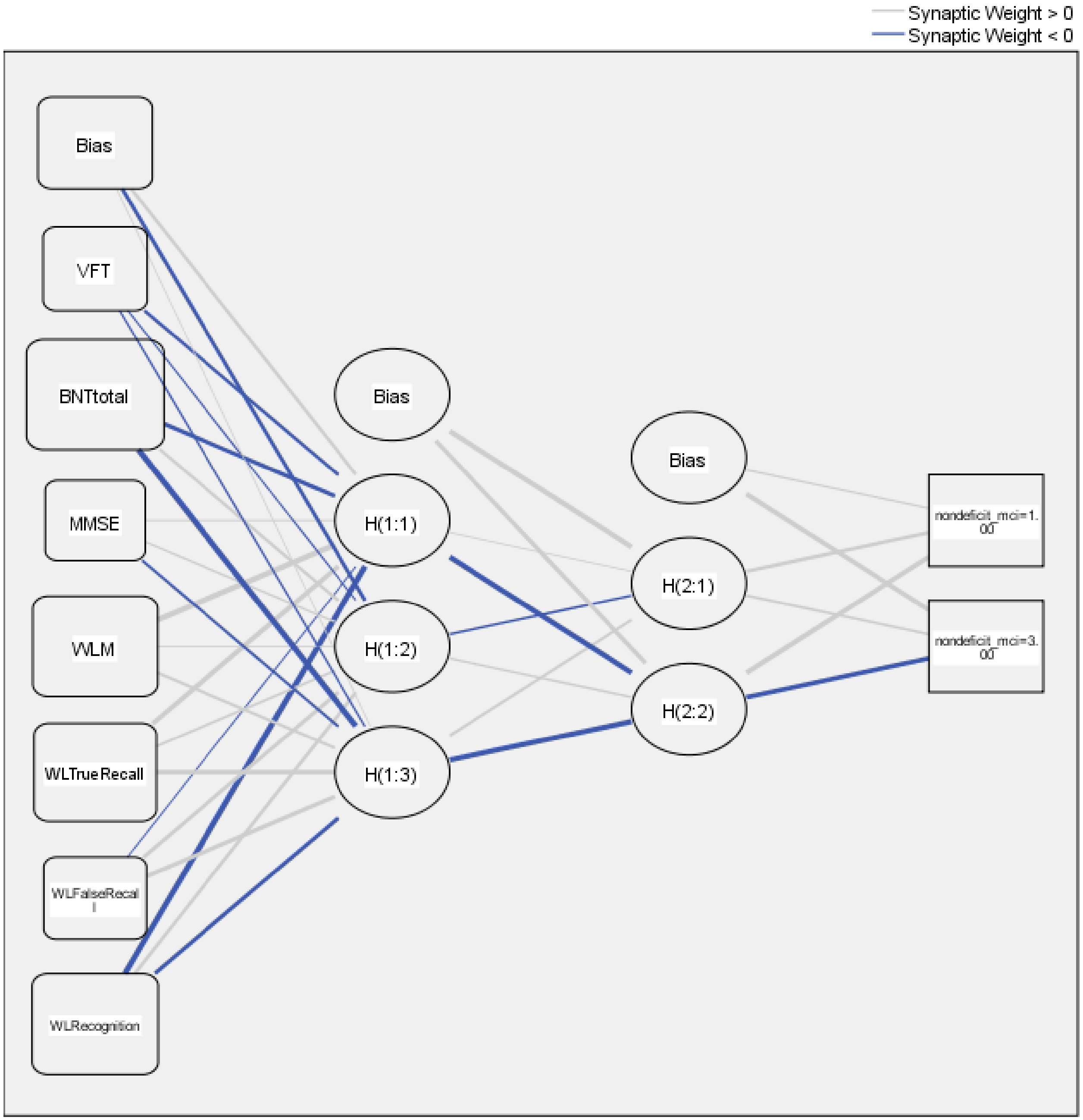

Hidden layer activation function: Hyperbolic tangent

Output layer activation function: Identity

Model Summary

Training	Sum of Squares Error	6.176
	Percent Incorrect Predictions	23.9%
	Stopping Rule Used	1 consecutive step(s) with no decrease in error[a]
	Training Time	0:00:00.00
Testing	Sum of Squares Error	2.246
	Percent Incorrect Predictions	18.2%
Holdout	Percent Incorrect Predictions	25.8%

Dependent Variable: nondeficit_mci

a. Error computations are based on the testing sample.

Parameter Estimates

Predictor		Predicted						
		Hidden Layer 1			Hidden Layer 2		Output Layer	
		H(1:1)	H(1:2)	H(1:3)	H(2:1)	H(2:2)	[nondeficit_mci=1.00]	[nondeficit_mci=3.00]
Input Layer	(Bias)	.313	-.362	.008				
	VFT	-.293	-.077	-.134				
	BNTtotal	-.417	.287	-1.064				
	MMSE	.069	.198	-.222				
	WLM	.777	.062	.265				
	WLTrueRecall	.558	.211	.465				
	WLFalseRecall	-.062	.394	.424				
	WLRecognition	-.799	.323	-.415				
Hidden Layer 1	(Bias)				.699	.441		
	H(1:1)				.030	-.736		
	H(1:2)				-.200	.199		
	H(1:3)				.252	-.750		
Hidden Layer 2	(Bias)						.118	.537
	H(2:1)						.327	.221
	H(2:2)						.565	-.518

Independent Variable Importance

	Importance	Normalized Importance
VFT	.071	27.4%
BNTtotal	.259	100.0%
MMSE	.048	18.5%
WLM	.189	72.8%
WL True Recall	.174	67.0%
WL False Recall	.063	24.4%
WLRecognition	.196	75.4%
